# Gluteal hemangioma mimicking a neuroendocrine tumor: pitfalls in neuroendocrine tumor diagnostic testing

**DOI:** 10.1210/jcemcr/luag025

**Published:** 2026-02-27

**Authors:** Glenn D Braunstein, Elie M Gindi, David J Kanani, Earl Brien, George R Matcuk, Farres Obeidin

**Affiliations:** Department of Medicine, Cedars-Sinai Medical Center, Los Angeles, CA 90048, USA; Department of Medicine, Cedars-Sinai Medical Center, Los Angeles, CA 90048, USA; Department of Medicine, Cedars-Sinai Medical Center, Los Angeles, CA 90048, USA; Department of Surgery, Cedars-Sinai Medical Center, Los Angeles, CA 90048, USA; Department of Imaging, Cedars-Sinai Medical Center, Los Angeles, CA 90048, USA; Department of Pathology & Laboratory Medicine, David Geffen School of Medicine at UCLA, Los Angeles, CA 90095, USA

**Keywords:** neuroendocrine tumors, somatostatin receptor, ^68^Ga-DOTATATE PET scan, hemangioma, gluteus maximus

## Abstract

Somatostatin receptor scanning with radiolabeled somatostatin analogues is an important modality for localizing neuroendocrine tumors (NETs) but may also show activity in normal tissues and non-NET pathologies. A patient with a history of a parathyroid adenoma, papillary thyroid carcinoma, and acoustic neuroma was suspected of harboring a NET based upon 2 circulating tumor nucleic acid tests that used different methodology and a positive ^68^gallium-dodecanetetraacetic acid-tyrosine-3-octreotate (^68^Ga-DOTATATE) scan corresponding to a 2.2-cm right gluteal mass, which was found to be an intramuscular hemangioma expressing somatostatin receptor 2 subtype (SSTR2) in the endothelial cells. Hemangiomas are among the most frequently reported lesions mimicking a NET and should be considered when somatostatin receptor scanning localizes an isolated lesion in an unusual anatomic area.

## Introduction

Neuroendocrine tumors (NETs) most commonly are located in the gastrointestinal tract, pancreas, or lung, but also encompass other tumors arising from neuroendocrine cells widely distributed throughout the body. They may elicit symptoms from local growth or secretion of hormones or may remain asymptomatic and be incidentally discovered [1]. NETs have high concentrations of somatostatin receptors, which allows localization and staging with radiolabeled somatostatin analogues such as the positron-emitting ^68^gallium-dodecanetetraacetic acid-tyrosine-3-octreotate (^68^Ga-DOTATATE), which has high affinity binding to the somatostatin receptor 2 subtype (SSTR2) [2, 3]. Although highly sensitive for detecting NETs, ^68^Ga-DOTATATE is not specific for NET, since SSTR2s are present in many normal tissues and neoplasms, inflammation, or other pathological processes, and those tissues may exhibit ^68^Ga-DOTATATE uptake [2-16]. An example is the patient reported below who was found to have a gluteal hemangioma expressing SSTR2 that had high ^68^Ga-DOTATATE avidity.

## Case presentation

A 60-year-old man underwent an evaluation for a possible NET based upon his past history and the finding of a positive primary signal for a NET on a blood multicancer early detection test that measures methylation signatures in cell-free deoxyribonucleic acid (DNA) (Galleri test; GRAIL, Menlo Park, CA 94025). This test has an overall sensitivity for NET of 83.3% (23.1% for stage I, 71.4% stage II, 93.3% stage III, 100% stage IV), and a specificity of 99.5% [17].

At age 41 years, he had a right lower parathyroid adenoma removed because of hyperparathyroidism. During the surgery, a papillary thyroid carcinoma was found, and he underwent a total thyroidectomy, which revealed a multifocal papillary carcinoma metastatic to perithyroidal and central compartment lymph nodes (stage 1 tumor). He received 144 mCi of radioactive iodine and had no evidence of recurrence based upon yearly undetectable serum thyroglobulin and neck ultrasounds. A left vestibular schwannoma was found at age 52 and was successfully treated with CyberKnife radiosurgery. Due to his history and age, he elected to have the multicancer early detection test noted above.

He did not have a family history of multiple endocrine neoplasia or von Hippel-Lindau disease. His brother has spinocerebellar ataxia, and the patient is a carrier for the type 17 gene. The patient had undergone a normal colonoscopy and upper gastrointestinal endoscopy within the year. Prior abdominal computed tomography (CT) scans demonstrated stable hepatic hemangiomas.

## Diagnostic assessment

Physical examination was normal, as were laboratory tests for hematologic, renal, hepatic, and electrolyte abnormalities. A chromogranin A was slightly elevated but returned to normal after a proton-pump inhibitor was discontinued. A ribonucleic acid (RNA)-based blood test that is designed to examine 51 genes related to NETs with a 91.9% sensitivity and 84.98% specificity was positive for a NET with a low risk of progression (NETest 2.0, Wren Laboratories, Branford, CT) [18]. Genetic testing for familial forms of NET was negative (performed at Invitae Corporation, San Francisco, CA). The genes examined included cyclin dependent kinase inhibitor 1B (*CDKN1B*), multiple endocrine neoplasia type 1 (*MEN1*), rearranged during transfection (*RET*), succinate dehydrogenase assembly factor 2 (*SDHAF2*), succinate dehydrogenase subunit B (*SDHB*), succinate dehydrogenase subunit C (*SDHC*), succinate dehydrogenase subunit D (*SDHD*), tuberous sclerosis complex 1 (*TSC1*), tuberous sclerosis complex 2 (*TSC2*), and von Hippel-Lindau (*VHL*). A capsule endoscopic examination of his gastrointestinal tract did not reveal any abnormalities.

**Figure 1:**
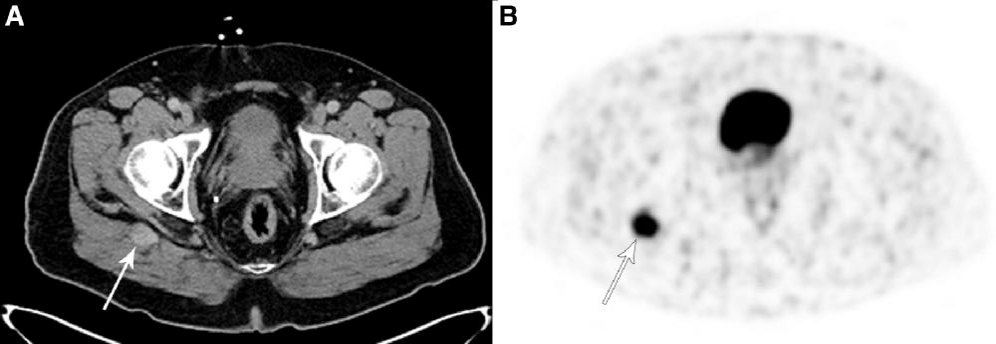
(A) Non-contrast CT scan taken at time of PET scan for attenuation correction to identify anatomic location of ^68^Ga-DOTATATE radiopharmaceutical. A 2.2-cm mass is present deep within the right gluteus maximus muscle (arrow); (B) ^68^Ga-DOTATATE PET scan showing intense (SUV = 11.2) uptake in the mass (arrow).

**Figure 2:**
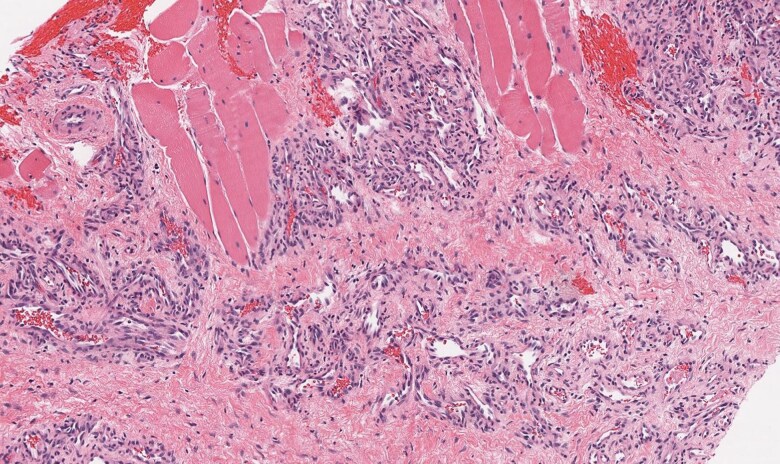
Core biopsy demonstrated a lobulated, benign vascular proliferation with associated fibrosis arising within the skeletal muscle (100× magnification).

**Figure 3:**
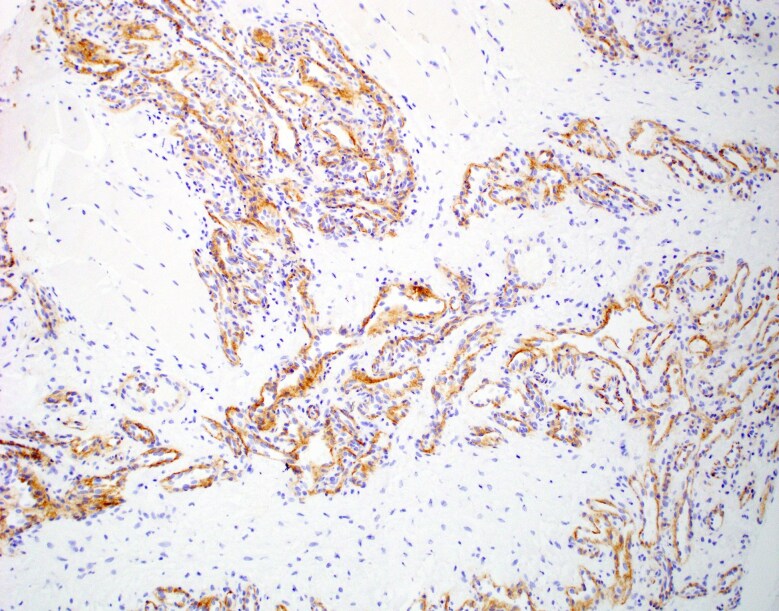
SSTR2 immunohistochemical stain showing cytoplasmic positivity in the endothelial cells (100× magnification). The SSTR2 used for this study was the EP149 rabbit monoclonal antibody from Cell Marque (Rocklin, CA).

A total body CT scan with contrast revealed a 2.2-cm enhancing mass deep in the right gluteal maximus muscle (Fig. 1A), which was not seen on a non-contrast abdominal/pelvic CT scan performed 5 ½ years before. A ^68^Ga-DOTATATE positron emission tomography (PET) scan demonstrated marked uptake in the lesion with a standardized uptake value (SUV) of 11.2 (Fig. 1B), indicating a high concentration of SSTR2 [2]. A CT-guided biopsy of the right buttock lesion was not diagnostic, showing only skeletal muscle and perimysial adipose tissue. Three months later, an attempted open excision of the lesion revealed an unencapsulated mass of small blood vessels surrounding larger vessels and nerves. Due to extensive bleeding, the surgeon cauterized the small vessels but was unable to obtain tissue for pathologic exam. Two and a half months later, a magnetic resonance imaging scan (MRI) of the pelvis demonstrated a tissue relaxation time-2 weighted image (T2) hyperintense mass that was slightly tissue relaxation time-1 weighted image (T1) hyperintense to muscle deep in the gluteus maximus muscle which was slightly enlarged over the initial measurements. Four months following the surgery, he underwent a technetium-99m-radiolabeled red cell scan which showed focal blood pool activity in the right gluteal lesion compatible with hemangioma. A repeat Galleri study was no longer positive for a NET. Nevertheless, because the gluteal uptake on the hemangioma scan could have been due to a vascular tumor, the slight increase in the size of the mass, and the lack of visualization of the lesion on the non-contrast CT scan 5 ½ years before, the patient had a repeat imaging-directed core needle biopsy of the lesion. The pathology confirmed an intramuscular hemangioma (Fig. 2), with intense SSTR2 immunohistochemical staining of the cytoplasm in the endothelial cells (Fig. 3).

## Treatment

Because the lesion was determined to be a benign intramuscular hemangioma, no further interventional therapies were deemed necessary.

## Outcome and follow-up

The patient remains well one year following the initial discovery of the gluteal hemangioma, which has increased in maximum diameter by 0.6 mm on a recent CT scan.

## Discussion

The majority of NETs express SSTR2, which makes ^68^Ga-DOTATATE an excellent imaging agent for their localization and monitoring because of the high affinity and specificity of DOTATATE for that SSTR subtype [2, 5, 16]. However, many normal tissues also express SSTR2, with the highest physiological ^68^Ga-DOTATATE uptake in the spleen, adrenal glands, kidneys, and pituitary, as well as moderate uptake in the liver, salivary glands, and thyroid [2]. Indeed, there is a significant correlation between the ^68^Ga-DOTATATE SUVs and SSTR2 concentration measured by reverse transcriptase polymerase chain reaction [2].

In addition to normal tissues and NETs, SSTR2 is expressed in macrophages, peripheral blood mononuclear cells, osteoblasts during endochondral bone formation, and growing vascular endothelial cells [19-21]. Thus, it is not surprising that inflammatory conditions, various neoplasms, osteoblast activation, and vascular abnormalities may express SSTR2, and, hence, attract the ^68^Ga-DOTATATE isotope. Table 1 lists conditions besides NETs which have demonstrated ^68^Ga-DOTATATE uptake on positron emission tomography scans [3-16]. Of interest, one of the most frequently reported “false positive” ^68^Ga-DOTATATE PET/CT scans is hemangiomas, especially vertebral hemangiomas [3, 8-10, 12, 15, 16].

**Table 1:** Positive ^68^Ga-DOTATATE scans in conditions other than neuroendocrine tumors

**Anatomic variants**
Pancreatic uncinate process
Splenosis
**Inflammatory conditions**
Atherosclerotic lesions
Chronic thyroiditis
Pancreatitis
Prostatitis
Reactive lymph nodes
**Neoplasms**
Breast carcinoma
Elastofibroma
Hepatocellular carcinoma
Gastric carcinoma
Lung cancer
Meningioma
Mesenchymal oncogenic osteomalacia tumors
Non-Hodgkin lymphoma
Osteosarcoma
Ovarian carcinoma
Parathyroid adenoma
Parotid adenoma
Plasmacytoma
Prostate carcinoma
Renal cell carcinoma
Small cell carcinoma
Squamous cell carcinoma
Thyroid carcinoma (non-medullary)
Uterine leiomyoma
Vestibular schwannoma
**Osteoblastic activity**
Degenerative joint disease
Epiphyseal growth plate
Fibrous dysplasia
Fracture
Tibial enchondroma
**Vascular lesions**
Epitheloid hemangioendothelioma
Hemangioblastomas
Hemangioma
Vertebral
Interosseous
Splenic
Cardiac
Primary nodal
Anastomosing

Although hemangiomas are common, their location in skeletal muscle is rare, accounting for approximately 0.8% of benign blood vessel tumors [22]. Of these, about 3% are located in the buttock's region [23, 24], and to our knowledge, none have been reported to have uptake by a somatostatin analogue imaging agent.

The 2 tests that were used to identify circulating tumor DNA or RNA were both positive for a NET cell of origin. Although there is no evidence that immunohistochemical expression of SSTR2 correlates with the abnormalities in DNA methylation in the Galleri test or the NET-associated RNA in the NETest®, it is possible that both were detecting signals of SSTR2 overexpression emanating from the gluteal hemangioma. Since data from patients with known hemangiomas but without a NET have not been published for these tests, this assumption remains speculative. The reason that the second Galleri test was no longer positive with a NET cancer signal of origin is unclear but may be the result of the extensive cauterization of the vascular lesion, which could have reduced the amount of abnormal DNA entering the circulation to a level below the limits of detection in the assay.

Because an isolated NET located deep within the gluteus maximus muscle has not been described in the literature nor encountered by several colleagues with extensive experience with NETs, our patient underwent extensive testing to find a primary NET that may have been the source of a gluteal skeletal muscle metastasis. Neither the initial image-guided biopsy of the lesion nor the open surgical procedure yielded a diagnosis, although the marked vascularity encountered during the surgery raised the possibility that this represented a SSTR2-expressing hemangioma, which was confirmed with the later biopsy. Our patient serves as a good reminder that lesions beside NETs can express SSTR2 and be detected by tests designed for NET detection and localization, and that SSTR2 expression alone does not necessarily reflect true neuroendocrine tumor biology

## Learning points

- Hemangiomas may express large quantities of somatostatin subtype 2 receptors.
- Somatostatin analogue radiopharmaceuticals may localize in some hemangiomas.
- In addition to hemangiomas and other vascular lesions, some inflammatory and neoplastic processes may express sufficient somatostatin receptors to be detected by imaging with somatostatin analogue radiopharmaceuticals.

## Data Availability

Data sharing is not applicable to this article as no datasets were generated or analyzed during the current study.

## Abbreviations

CT: computed tomography
^68^Ga-DOTATATE: ^68^gallium-dodecanetetraacetic acid-tyrosine-3-octreotate
NET: neuroendocrine tumor
PET: positron emission tomography
SSTR2: somatostatin receptor 2 subtype
SUV: standardized uptake value
